# Identification of G Protein-Coupled Receptors (GPCRs) in Primary Cilia and Their Possible Involvement in Body Weight Control

**DOI:** 10.1371/journal.pone.0128422

**Published:** 2015-06-08

**Authors:** Yoshihiro Omori, Taro Chaya, Satoyo Yoshida, Shoichi Irie, Toshinori Tsujii, Takahisa Furukawa

**Affiliations:** 1 Laboratory for Molecular and Developmental Biology, Institute for Protein Research, Osaka University, 3-2 Yamadaoka, Suita, Osaka, Japan; 2 JST, PRESTO, 3-2 Yamadaoka, Suita, Osaka, Japan; Osaka University Graduate School of Medicine, JAPAN

## Abstract

Primary cilia are sensory organelles that harbor various receptors such as G protein-coupled receptors (GPCRs). We analyzed subcellular localization of 138 non-odorant GPCRs. We transfected GPCR expression vectors into NIH3T3 cells, induced ciliogenesis by serum starvation, and observed subcellular localization of GPCRs by immunofluorescent staining. We found that several GPCRs whose ligands are involved in feeding behavior, including prolactin-releasing hormone receptor (PRLHR), neuropeptide FF receptor 1 (NPFFR1), and neuromedin U receptor 1 (NMUR1), localized to the primary cilia. In addition, we found that a short form of dopamine receptor D2 (DRD2S) is efficiently transported to the primary cilia, while a long form of dopamine receptor D2 (DRD2L) is rarely transported to the primary cilia. Using an anti-Prlhr antibody, we found that Prlhr localized to the cilia on the surface of the third ventricle in the vicinity of the hypothalamic periventricular nucleus. We generated the *Npy2r-Cre* transgenic mouse line in which Cre-recombinase is expressed under the control of the promoter of *Npy2r* encoding a ciliary GPCR. By mating *Npy2r-Cre* mice with *Ift80* flox mice, we generated *Ift80* conditional knockout (CKO) mice in which Npy2r-positive cilia were diminished in number. We found that *Ift80* CKO mice exhibited a body weight increase. Our results suggest that Npy2r-positive cilia are important for body weight control.

## Introduction

The primary cilia are hair-like organelles that project out from the cell surface in various types of cells. In humans, dysfunction of cilia causes a broad range of overlapping clinical phenotypes termed “ciliopathies,” which include retinal degeneration, polycystic kidney disease, polydactyly, and obesity [1,2]. In certain ciliopathies, including Bardet-Biedl syndrome (BBS) and Alström syndrome, patients exhibit hyperphagia, obesity, and diabetes [2]. Loss of neuronal cilia in the central nervous system (CNS) causes defects in feeding behavior and obesity, suggesting that ciliary function in the CNS is essential for body weight control and energy homeostasis [3]. However, the precise molecular mechanisms underlying the pathogenesis of obesity in ciliopathies remain unclear.

Cilia function as signaling hubs by harboring membrane receptors including G protein-coupled receptors (GPCRs), which transduce extracellular signals into cellular responses [4,5]. For example, in the nose, olfactory receptors on the cilia of olfactory cells in the nasal cavity detect odors and initiate signaling cascades in olfactory neurons. In the retina, photoreceptor outer segments, which are evolutionarily highly modified primary cilia, contain light-sensitive GPCRs, known as opsins, to sense light [6].

Several GPCRs, including somatostatin receptor 3 (SSTR3), serotonin receptor 6 (5HTR6), melanin-concentration hormone receptor 1 (MCHR1) and dopamine receptors (DRD1, DRD2 and DRD5), are known to localize to neuronal cilia [7–11]. Although the biological significance of the localization of these GPCRs in the neuronal primary cilia is still unclear, several studies have suggested the necessity of localization of these GPCRs in neuronal primary cilia. For example, *Sstr3*-deficient mice showed impairment of object recognition and hippocampal LTP [12]. *Mchr1*-deficient mice exhibited cognitive deficits and alterations of NMDA receptor function [13].

The human genome contains more than 800 genes encoding GPCRs, which constitute the largest family among transmembrane receptors [14]. Odorant receptors form a large family of ciliary GPCRs in the vertebrate genome. Although the natural ligands for approximately 210 non-odorant GPCRs have been identified, endogenous ligands are unknown for approximately 150 non-odorant GPCRs, which are called “orphan GPCRs”.

Since we hypothesized that there still remain uncharacterized GPCRs that localize to cilia and have important biological functions, we performed a screen to identify ciliary GPCRs. We analyzed the subcellular localization of 138 non-odorant human or mouse GPCRs. We found that prolactin-releasing hormone receptor (PRLHR), neuropeptide FF receptor 1 (NPFFR1), and neuromedin U receptor 1 (NMUR1) localize to cilia. We also found that the short isoform of dopamine receptor D2 (DRD2S) is efficiently transported to primary cilia while the long form (DRD2L) is not. Furthermore, we generated mutant mice that exhibited a ciliary defect in cells expressing a GPCR, Npy2r, and observed a significant body weight increase in these mice. Our results suggest that Npy2r-positive cilia play a significant role in proper body weight control.

## Materials and Methods

### Animal care

All procedures conformed to the ARVO statement for the Use of Animals in Ophthalmic and Vision Research, and these procedures were approved by the Institutional Safety Committee on Recombinant DNA Experiments (approval ID: 3380–4) and Animal Experimental Committees of the Institute for Protein Research (approval ID: 24-05-1), Osaka University, and were performed in compliance with the Institutional guidelines. Mice were housed in a temperature-controlled room at 22°C with a 12 h light/dark cycle. Fresh water and rodent diet were available at all times. Mice were sacrificed with carbon dioxide, and all efforts were made to minimize suffering.

### Antibodies

An antibody against mouse Prlhr or Npy2r was obtained by immunizing rabbits with the synthetic peptide MTSLSTETTGDPDLSSGC (1–17, N-terminal of Prlhr) or CVKKNNGPTDSFSEATNV (365–381, C-terminal of Npy2r), which was coupled to the carrier protein keyhole limpet hemocyanin. The antibody titer in the serum was measured by ELISA on plates coated with the synthetic peptide. The anti-peptide antibodies were affinity purified using a synthetic peptide-sepharose column.

### Construction of BAC-Npy2r-Cre transgenic mice

A BAC clone (clone ID: RP23-280I12) containing the entire *Npy2r* gene was purchased from the BACPAC Resource Center at the Children's Hospital Oakland Research Institute (Oakland, CA, USA). Two homologous arms (5’ arm, 1053 bp; 3’ arm, 1152 bp) from the *Npy2r* gene were amplified by PCR with the BAC clone. The PCR primers used were: 5’ TTGGCGCGCCCAACACCTCTGCACAAGGTTCCAT3’, 5’AAG CGGCCGCAACCAGTTCACTCTCACTTGGCCTG3’ for the 5’ homology arm and 5’ AATTAATTAACTGAAGATGGGCCCGGTAGGTGCAGA3’, 5’AAGGCCGGCCT TACACATTGGTAGCCTCCGAAAA3’ for the 3’ homology arm. Two homologous arms were inserted into both sides of the *Cre-pA* cassette in the pLD53-SCA-Cre-B shuttle vector [15]. The *Cre-pA* cassette was introduced into the 5’ UTR position in the second exon of the *Npy2r* gene by homologous recombination [16]. We confirmed the correct insertion of the *Cre* gene into the *Npy2r* locus by sequencing.

### Generation of BAC-Npy2r-Cre transgenic mice and *Ift80-Npy2r* CKO mice

The entire BAC-Npy2r-Cre transgene was purified as previously described [15]. The purified BAC-Npy2r-Cre construct was injected into the pronuclei of fertilized one-cell eggs of B6C3F1 mice (Japan SLC) followed by implantation into pseudopregnant foster mothers (ICR; Japan SLC). An ES clone, *Ift80* ^tm1a(KOMP)Wtsi^-IFT80_D09 (JM8A1.N3), in which the LacZ, neomycin-resistant (Neo) gene cassettes and *loxP* sites were inserted in the genomic region of *Ift80*, was purchased from the NIH Knock-Out Mouse Project (KOMP) repository. The ES cells were microinjected into C57BL/6 blastocysts to yield chimeric mice. These chimeric mice were crossed with C57BL/6 mice to generate *Ift80* flox mice. Heterozygous *Ift80* ^tm1a(KOMP)Wtsi^ mice were crossed with B6-Tg(CAG-FLPe)37 mice (#RBRC01835, RIKEN BRC) to remove the *FRT*-flanked *LacZ* and *Neo* cassettes by Flp recombinase. We then mated the BAC-Npy2r-Cre mouse line with the *Ift80* flox mouse line to establish an *Ift80-Npy2r-Cre* CKO (*IFT80* ^*flox/flox*^; *Npy2r-Cre* ^+^) mouse line. We used *Ift80* ^*flox/flox*^; *Npy2r-Cre* ^-^ or *Ift80* ^*flox/+*^; *Npy2r-Cre* ^-^ mice as control mice. *Ift80-Npy2r-Cre* CKO mice have a mixed C57BL/6 and B6C3HF1 background. To observe Cre activities in the BAC-Npy2r-Cre transgenic mice, we crossed them with the *Rosa26-mTFP1* reporter strain (#RBRC05147, RIKEN BRC) [17] and analyzed *mTFP1* expression.

### Cell culture and transfection

NIH3T3 cells [18] were grown in Dulbecco's Modified Eagle Medium (DMEM; Sigma) with 10% fetal calf serum and 2 mg/L L-glutamine. Transfection was performed using Lipofectamine-LTX (Invitrogen) according to the manufacturer’s instructions. We transfected 2.5 μg of DNA with 5 μl of Lipofectamine-LTX into NIH3T3 cells cultured in a 35-mm dish. At 24 hrs after transfection, the medium was replaced by serum-free DMEM. Cells were cultured for 24 hrs in serum-free medium to induce ciliogenesis. For immunostaining, cells were washed with phosphate-buffered saline (PBS), fixed with 4% paraformaldehyde in PBS for 10 min at room temperature and subsequently incubated with blocking solution (5% normal goat serum and 0.1% Triton-X100 in PBS) for 30 min. Cells were immunostained with a primary antibody in the blocking solution for 4 hrs at room temperature and subsequently incubated with the secondary antibody solution for 2 hrs at room temperature. We classified ciliary GPCRs and non-ciliary GPCRs by counting the number of ciliated cells with the FLAG signal in the cilia out of 20 ciliated cells. When we observed more than 14 ciliated cells with the FLAG signal in the cilia out of 20 ciliated cells, we classified them as ciliary GPCRs (S1 Table).

### Immunohistochemical analysis of brain tissue

Perfusion-fixed brains were postfixed in 4% paraformaldehyde in PBS for overnight at 4°C, embedded in TissueTec OCT compound 4583 (Sakura), frozen and sectioned. Frozen 20 μm sections on slides were dried for 30 min at room temperature, rehydrated in PBS for 5 min, incubated with blocking solution (5% normal goat serum, and 0.5% Triton X-100 in PBS) for 1 hr, and then with primary antibodies for 4 hrs at room temperature. Slides were washed with PBS three times for 10 min each time and incubated with secondary antibodies for 2 hrs at room temperature. The specimens were observed under a laser confocal microscope (LSM700, Carl Zeiss). For preabsorption, antibodies were first incubated with a 20-fold molar excess of peptides in blocking solution at room temperature for 4 hrs and used for immunostaining. Co-immunostaining of cilia with antibodies against Adcy3 and Npy2r was performed using a Zenon Labeling Kit (Lifetechnologies) as described previously [19]. We used the following primary antibodies: mouse monoclonal antibodies specific to acetylated α-tubulin (1:1000, Sigma), FLAG-M2 (1:1000, Sigma), GFAP (1:500, Sigma), and Arl13b (1:1000, NeuroMab); and rabbit polyclonal antibodies to FLAG (1:1000, Sigma), Prlhr (1:300) and Npy2r (1:500); and a rat monoclonal antibody to GFP (1:2000, nacalai). Nuclei were stained with DAPI. We used Cy3-conjugated secondary antibodies (1:400, Jackson ImmunoResearch Laboratories) and Alexa Fluor 488-conjugated secondary antibodies (1:400, Sigma).

### Quantitative real-time PCR (Q-PCR)

Q-PCR was performed using SYBR Green ER Q-PCR Super Mix (Invitrogen) and Thermal Cycler Dice Real Time System Single MRQ TP870 (Takara) according to the manufacturers’ protocols. Quantification was performed by Thermal Cycler Dice Real Time System software Ver. 2.0 (Takara). Primer sequences were as follows: for *Npy2r*, forward (F), 5’-CTCATCTGAGAAGGAACGCGCAAG-3’ (*Npy2r-F1*), and reverse (R), 5’-CAGGACTGTCTCCAGACTGGCTGT-3’ (*Npy2r-R2*); *GAPDH*, F, 5’-ACTGGCATGGCCTTCCGTGTTCCTA-3’ (*GAPDH-P1*), and R, 5’-TCAGTGTAGCCCAAGATGCCCTTC-3’ (*GAPDH-P2*).

### Western blot analysis

Transfected HEK293 cells were washed by PBS twice and lysed in SDS-sample buffer. Western blot analysis was performed using a semidry transfer cell (Bio-Rad) with PVDF membrane. Signals were detected using Pierce Western Blotting Substrate Plus (Thermo). We used the following primary antibodies: mouse monoclonal antibodies specific to FLAG (M2, 1:6000, Sigma) and α-tubulin (DM1A, 1:6000, Sigma).

### Statistical analysis

Data are presented as means ± SD. Statistical significance was evaluated using the Student’s *t* test, and *p* < 0.03 was taken to be statistically significant.

## Results and Discussion

### Screen for cilia-localized GPCRs

**Fig 1:**
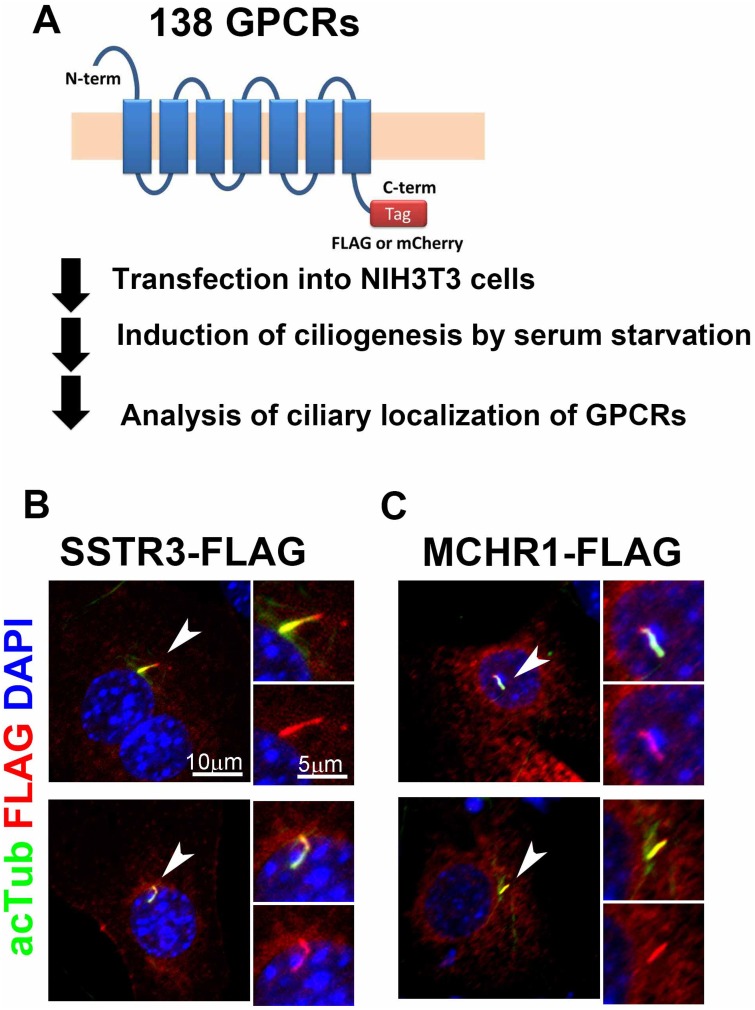
A screen to identify ciliary GPCRs using NIH3T3 cells. **A**) Strategy of the screen to identify ciliary GPCRs. We constructed plasmids expressing 138 non-odorant GPCRs fused with a FLAG or mCherry tags at their C-terminals. These constructs were transfected into NIH3T3 cells. At 24 hrs after transfection, ciliogenesis was induced by serum starvation. At 48 hrs after transfection, subcellular localization of GPCRs was analyzed by immunostaining using an anti-FLAG or anti-mCherry antibody. Cilia were marked with an anti-acetylated α-tubulin antibody. **B, C**) Localization of FLAG-tagged Sstr3 (B) and MCHR1 (C) to cilia in NIH3T3 cells. GPCRs were stained with an anti-FLAG antibody (red) and cilia were stained with the anti-acetylated α-tubulin antibody (green). Co-localization of FLAG (red) and acetylated α-tubulin (green) signals was observed in cilia. Nuclei were stained with DAPI (blue). Arrowheads indicate cilia. Scale bars, 10 μm (**B, C**) and 5 μm (insets in **B, C**).

**Fig 2:**
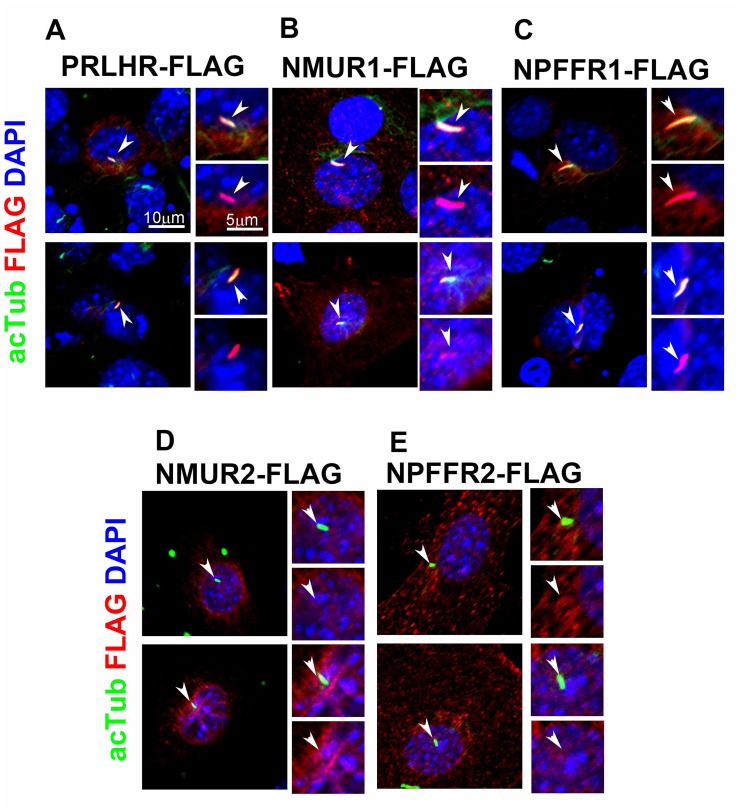
Newly identified GPCRs localized to cilia. **A–C**) Three GPCRs, PRLHR (A), NMUR1 (B) and NPFFR1 (C), were identified to localize to primary cilia. GPCRs were stained with the anti-FLAG antibody (red) and cilia were stained with the anti-acetylated α-tubulin antibody (green). Co-localization of FLAG (red) and acetylated α-tubulin (green) signals was observed in the cilia. **D, E)** NMUR2 (D) and Npffr2 (E), highly conserved paralogs of NMUR1 and NPFFR1, respectively, did not localize to cilia. GPCRs were stained with the anti-FLAG antibody (red) and cilia were stained with the anti-acetylated α-tubulin antibody (green). FLAG (red) and acetylated α-tubulin (green) signals were non-overlapped. Nuclei were stained with DAPI (blue). Arrowheads indicate cilia. Scale bars, 10 μm (A-E) and 5 μm (insets in A-E).

To identify previously unknown GPCRs that localize to primary cilia, we screened 138 non-odorant GPCRs for which the endogenous ligands had been identified (Fig 1A). We generated constructs expressing a FLAG- or mCherry-tagged full length GPCR under the control of the CAG promoter [20]. We transfected each of these constructs into NIH3T3 cells, induced ciliogenesis by serum starvation, and co-immunostained cilia using an anti-FLAG or anti-mCherry antibody together with an anti-acetylated α-tubulin antibody to label the ciliary axoneme. As a positive control, we used Sstr3 and Mchr1, which are known to localize to cilia in neurons in the mouse brain [8,21]. We observed that Sstr3 signals labeled with the anti-FLAG antibody in NIH3T3 cells co-localize with a ciliary signal immunostained by the anti-acetylated α-tubulin antibody (Fig 1B). Similarly, we confirmed that FLAG-tagged MCHR1 also localizes to cilia in NIH3T3 cells (Fig 1C). Using this system, we screened the subcellular localization of 138 GPCRs (S1 Table). In this screen, we identified for the first time three GPCRs that localize to cilia: prolactin releasing hormone receptor (PRLHR), neuromedin U receptor 1 (NMUR1), and neuropeptide FF receptor 1 (NPFFR1; Fig 2A–2C). NMUR1 and NPFFR1 have highly conserved paralogs, NMUR2 (42% amino acid identity) and Npffr2 (47% amino acid identity), respectively. We also examined subcellular localization of NMUR2 and Npffr2 in NIH3T3 cells. As far as we tested, localization of these proteins to primary cilia was not detected (Fig 2D and 2E). We measured the transfection efficiency of each ciliary GPCR in NIH3T3 cells by immunostaining analysis (S1A Fig). We found that approximately 5 to 10% of cells express FLAG-tagged GPCRs in NIH3T3 cell bodies. In addition, we transfected FLAG-tagged GPCR expression constructs into HEK293 cells and performed Western blot analysis. We confirmed that the approximate molecular size of each FLAG-tagged GPCR was consistent with a predicted molecular mass (S1B Fig), suggesting that full-length GPCRs were produced in HEK293 cells.

The RF-amide neuropeptide family molecules that possess an RF-amide motif at their C-termini function as peptide ligands for GPCRs [22]. Neuropeptide FF (NPFF) belongs to the RF-amide neuropeptide family and functions as a pain-modulating peptide with anti-opioid activity [23]. Intracerebroventricular (ICV) administration of NPFF induces gastrointestinal motility [24], reduces food intake [25,26], increases water intake [26], and causes hypothermia [27]. NPFFR1 has been identified as one of two NPFF-binding GPCRs [28]. NPFFR1 is highly expressed in the rat hypothalamus [22,28]. Prolactin-releasing hormone (PRLH), also known as prolactin-releasing peptide (PrRP), is an RF-amide neuropeptide family member. ICV injection of Prlh stimulates hypothalamic neurosecretion [29–31], elevates blood pressure [32], and reduces food intake [33]. Since ICV injection of Prlh increases body temperature and oxygen consumption in rats, the Prlh effect on energy homeostasis is thought to be mediated through increased energy expenditure [34]. Prlh was thought to act as a hypothalamic releasing factor for prolactin secretion in the rat pituitary gland [35], however, Prlh was not detected in the external layer of the median eminence in the hypothalamus [36], suggesting that Prlh is not a classic hypothalamic releasing factor, at least in rodents. ICV administration of PRLH induces secretion of prolactin as well as other pituitary hormones, including ACTH, oxytocin, vasopressin, and luteinizing hormone. PRLH has also been proposed to play roles in stress responses, waking/sleeping states and analgesia [37,38]. PRLHR, also known as GPR10, was identified as a receptor for PRLH [35]. *Prlhr*-knockout mice and *Prlhr*-null rats (OLETF rats) exhibited obesity and hyperphagia [39–41], suggesting an essential function of Prlhr in body weight control, feeding behavior and energy homeostasis. Our observation that PRLHR and NPFFR1 localize to cilia in the cultured cell system suggests the possibility of ciliary localization of these proteins in neuronal tissues.

Nmur1 is a GPCR which interacts with two physiologically active peptides, neuromedin U (NMU) [42] and neuromedin S (NMS) [43]. NMU is a brain-gut neuropeptide that has a potent activity to cause uterine smooth muscle contraction [44]. ICV injection of NMU suppresses food intake [42,45] and energy homeostasis [46]. NMUR1 and NMUR2 have been identified as NMU receptors [42]. *Nmu*-null mutant mice develop obesity. NMU regulates feeding behavior and energy metabolism independent of the leptin signaling pathway [47]. Human and rat *Nmur1* are expressed in various tissues, including the pancreas and small intestine. Rat *Nmur2* is mainly expressed in specific regions of the brain, including the paraventricular nucleus (PVN) [42]. Our finding that NMUR1 localizes to cilia in NIH3T3 cells suggests the possibility that NMUR1 has a ciliary function in various tissues.

Although we classified GPCRs as either ciliary or non-ciliary GPCR in this screen using NIH3T3 cells, we cannot exclude the possibility that non-ciliary GPCRs identified in this screen may localize to cilia in other types of cells, because certain GPCR may require specific component(s) unexpressed in NIH3T3 cells for ciliary localization. For example, the olfactory CNG channel requires the CNGB1b protein for ciliary localization [48].

### The short isoform of dopamine D2 receptor efficiently localizes to cilia

**Fig 3:**
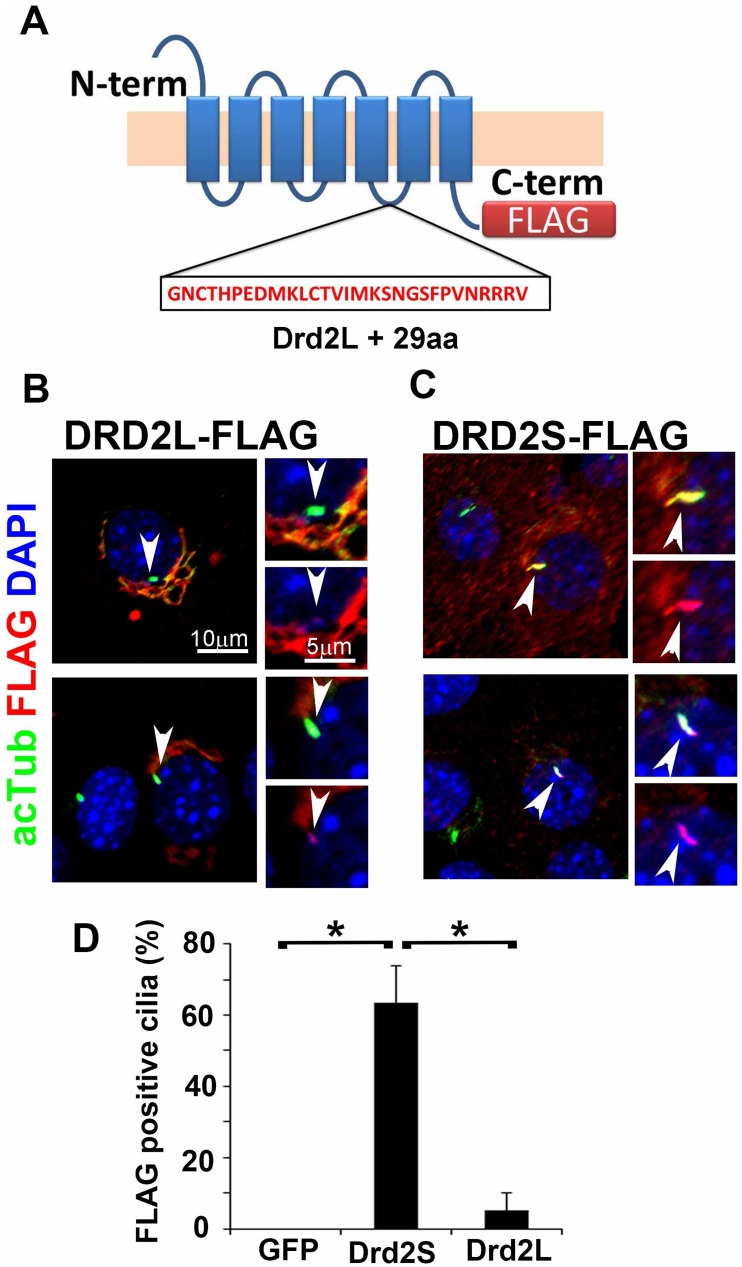
A short isoform of dopamine D2 receptor (DRD2S) efficiently localizes to cilia. **A**) Schematic diagrams of DRD2 isoforms, DRD2S and DRD2L. DRD2L differs from DRD2S in the presence of a 29-amino-acid insertion in the third intracellular loop. **B–D**) Subcellular localization of DRD2 isoforms. FLAG-tagged DRD2L (B) or DRD2S (C) was expressed in the NIH3T3 cells. Cells were stained with the anti-acetylated α-tubulin (green) and anti-FLAG (red) antibodies. DRD2L was mainly accumulated in perinuclear Golgi apparatuses-like structures but was barely localized to the cilia (B). In contrast, DRD2S was clearly enriched in cilia (C). Ratios of localization of DRD2S and DRD2L to cilia in ciliated NIH3T3 cells were quantified (D). The average values from three independent transfection experiments are shown (*p<0.03). Nuclei were stained with DAPI (blue). Arrowheads indicate cilia. Scale bars, 10 μm (B, C) and 5 μm (insets in B, C).

Dopamine receptor D2 (Drd2) is essential for the regulation of diverse physiological functions, including locomotor activity control and peptide hormone synthesis [49–51]. Drd2 also regulates food intake and body weight control [49]. The *Drd2* gene encodes two molecularly distinct isoforms, *Drd2s* (short isoform) and *Drd2l* (long isoform) [52]. The Drd2l protein differs from the Drd2s protein in the presence of a 29-amino-acid insertion in the third intracellular loop (Fig 3A). Although Drd2l and Drd2s exhibit similar pharmacological characteristics, Drd2l acts at postsynaptic sites of neurons and Drd2s functions as a presynaptic autoreceptor [53]. The dopamine receptors DRD1, DRD2L, and DRD5 were previously reported to localize to cilia in NIH3T3 cells and rat neurons [10]. Consistent with this report, we observed that Drd1 and Drd5 localize to cilia in NIH3T3 cells (S1 Table). On the other hand, we rarely observed DRD2L signals in cilia in NIH3T3 cells, and DRD2L signals were often accumulated in the Golgi-like structures surrounding the nucleus (Fig 3B). We also expressed FLAG-tagged DRD2S in NIH3T3 cells and analyzed its subcellular localization. Interestingly, we found a distinct accumulation of DRD2S signals in cilia but not in the Golgi-like structures (Fig 3C). The FLAG-tagged DRD2S localized to cilia in 63% of ciliated NIH3T3 cells stained with an anti-acetylated α-tubulin antibody, whereas the FLAG-tagged DRD2L localized to cilia only in 5% of ciliated cells (Fig 3D). This result indicates that a difference of only 29 amino acids between the two DRD2 isoforms is enough to discriminate ciliary localization, suggesting the possibility that DRD2S has a function in neuronal primary cilia in addition to its presynaptic autoreceptor function.

### Prlhr localizes to cilia in the hypothalamic third ventricle

**Fig 4:**
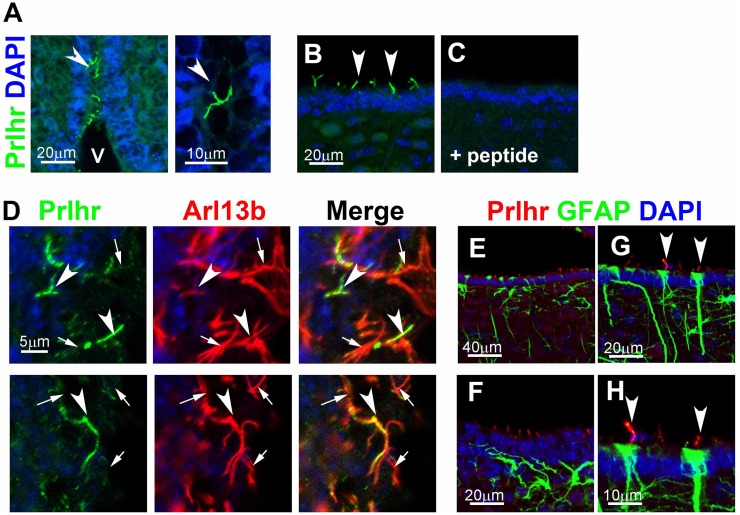
Prlhr localizes to cilia in the hypothalamic third ventricle. **A–D**) Prlhr was localized to the ventricular cilium in the adult mouse brain. Frozen sections containing the third ventricle of the adult mouse brain were immunostained with an anti-Prlhr antibody (green). The Prlhr signal was observed in cilia protruding from cells on the surface of the third ventricle (arrowheads in A, B, D). Preabsorption of the anti-Prlhr antibody with a synthetic Prlhr-peptide antigen abolished the Prlhr staining in the cilia (C). Cilia were coimmunostained with an anti-Arl13b antibody (D, red). Cilia with the Prlhr signal (arrowheads) were not clustered together, unlike the multiple cilia of ependymal cells that lacked the Prlhr signal (arrows). **E–H**) Ventricular Prlhr-positive cilia (red) were double stained with GFAP (green) which stains a subtype of tanycytes. A partial population of the GFAP-positive tanycytes (green) possesses Prlhr-positive cilia (arrowheads). Nuclei were stained with DAPI (blue). V: third ventricles. Scale bars, 20 μm (the left panel in A; B, C, F, G), 10 μm (the right panel in A; H), 5 μm (D), and 40 μm (E).

To investigate the subcellular localization of Prlhr in mouse tissues, we generated an antibody against mouse Prlhr. A pervious expression study of *Prlhr* by *in situ* hybridization showed that *Prlhr* mRNAs are expressed in the periventricular nucleus (PeVN) in the rat hypothalamus [54]. In the present study, we immunostained sections from the adult mouse brain using anti-Prlhr and anti-Arl13b antibodies. We observed Prlhr signals in cilia on the surface of the third ventricle around the PeVN (Fig 4A and 4B). Preabsorption of the anti-Prlhr antibody with a synthetic Prlhr-peptide antigen completely abolished the Prlhr staining in the cilia (Fig 4C). We also detected a colocalization of Prlhr and Arl13b signals in cilia (Fig 4D arrowheads). The Arl13b signal was also observed in multiple cilia of ependymal cells without the Prlhr signal (Fig 4D arrows). Because cilia with the Prlhr signal (arrowheads) were not clustered together, unlike the multiple cilia of ependymal cells that lacked the Prlhr signal (arrows), Prlhr cilia seem to extend from non-ependymal cells. A type of glial cells, called tanycytes, project few cilia on the surface of the third ventricle [55]. Recent studies have suggested potential roles of tanycytes in feeding behavior and energy balance [56]. We co-immunostained the hypothalamus using antibodies against GFAP, a tanycyte marker [56], and Prlhr. Although many of the Prlhr-positive cilia did not extend from GFAP-positive cells (Fig 4E and 4F), we found that at least a small number of Prlhr-positive cilia extended from GFAP-positive tanycytes with a long process extending into the parenchyma (Fig 4G and 4H). This result suggests that a partial population of tanycytes possesses Prlhr-positive cilia on the surface of the third ventricle.

### Ciliary defect in *Npy2r*-expressing cells causes a body weight increase in mice

**Fig 5:**
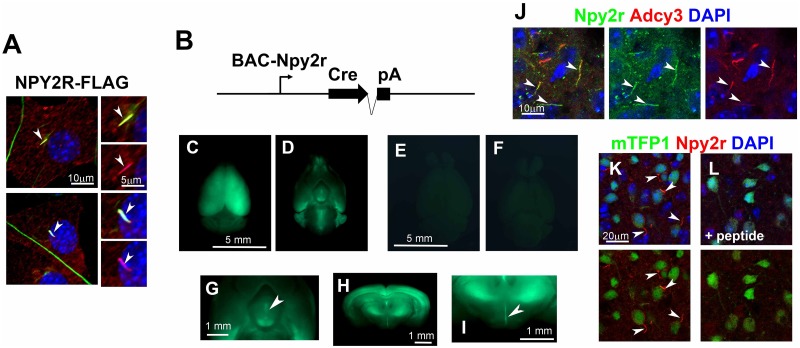
Subcellular localization of Npy2r and BAC-Npy2r-Cre transgenic mouse. **A)** The subcellular localization of Npy2r. FLAG-tagged Npy2r was expressed in the NIH3T3 cells. Cells were stained with the anti-acetylated α-tubulin (green) and anti-FLAG (red) antibodies. FLAG-tagged Npy2r signals were observed in NIH3T3 cells (red). Co-localization of Npy2r and acetylated α-tubulin signals was observed in cilia (arrowheads). Nuclei were stained with DAPI (blue). **B**) Diagram representing the BAC-Npy2r-Cre transgene construct. **C–L**) Expression of mTFP1 in the brains of *R26-CAG-LoxP-mTFP1*; *BAC-Npy2r-Cre* transgenic mice (C, D, G-I, K, L) and control mice (E, F). BAC-Npy2r-Cre transgenic mice were crossed with reporter mice, R26-CAG-LoxP-mTFP1, to detect the expression of the Cre recombinase in BAC-Npy2r-Cre transgenic mice. Dorsal (C, E), ventral (D, F, G) and coronal (H, I) views of the brain are shown. Broad expression of mTFP1 in the brain including the hippocampus, cerebral cortex, and hypothalamus (paraventricular nucleus, arrowheads in G, I) was observed. In the arcuate nucleus, Npy2r (green in J, arrowheads) colocalized with Adcy3 (a ciliary marker, red in J) in cilia. Npy2r-stained cilia (red) were often observed in mTFP1-positive cells (green in K). Preabsorption of the anti-Npy2r antibody with a synthetic Npy2r-peptide antigen abolished the Npy2r staining in the cilia (L). Scale bars, 10 μm (A, J), 5 μm (the inset in A), 5 mm (C–F), 1 mm (G–I), and 20 μm (K, L).

In our screen we found that Npy2r localizes to cilia in NIH3T3 cells, which is consistent with another recent report (Fig 5A) [57]. Neuropeptide Y (NPY) and its receptors are associated with hypothalamic regulation of feeding behavior, metabolism, and energy homeostasis in mammals [58,59]. *Npy2r*-deficient mice exhibited hyperphagia and excessive weight gain [60], suggesting an essential role of Npy2r in feeding behavior and body weight control. A recent study showed that conventional knockout mice lacking *BBIP10 (BBS18)*, encoding a component of the BBSome complex, exhibited obesity and a defect in the ciliary localization of Npy2r [57]. Although the ciliary localization of Npy2r is essential for the Npy2r signaling pathway in cells [57], it is unclear whether a ciliary defect in Npy2r-positive cells affects body weight control *in vivo*.

To investigate a possible ciliary function of *Npy2r*-expressing cells for normal body weight control, we generated mutant mice in which cilia are disrupted in cells expressing Npy2r. First, we generated BAC-Npy2r-Cre transgenic mice which express Cre recombinase under the control of the *Npy2r* promoter (Fig 5B). We mated BAC-Npy2r-Cre transgenic mice with the reporter mouse line, R26-CAG-LoxP-mTFP1 [17], in which mTFP1 expression is induced by the CAG promoter upon Cre expression. In these mice, we observed broad expression of mTFP1 in the brain, including the hippocampus, cerebral cortex, hypothalamic arcuate nucleus, and PVN (arrowheads) (Fig 5C, 5D, 5G, 5H and 5I). This mTFP1 reporter expression pattern induced in BAC-Npy2r-Cre transgenic mice is similar to the endogenous *Npy2r* expression pattern in the brain as previously reported [61]. In contrast, we observed almost complete loss of mTFP1 expression in the R26-CAG-LoxP-mTFP1 brain without the Cre allele (Fig 5E and 5F). In order to confirm the ciliary localization of Npy2r, we generated an anti-Npy2r antibody and co-immunostained the hypothalamic arcuate nucleus cells with antibodies against Npy2r and Adenylate cyclase 3 (Adcy3, a ciliary marker protein). We found that Npy2r colocalized with Adcy3 in cilia (Fig 5J). To confirm the overlapping expression pattern of mTFP1 and Npy2r in the hypothalamus, we immunostained the hypothalamus arcuate nucleus with the anti-Npy2r antibody. We found many Npy2r-stained cilia extending from mTFP1-positive cells in the hypothalamic arcuate nucleus of the *R26-CAG-LoxP-mTFP1;BAC-Npy2r-Cre* mice. (Fig 5K). Preabsorption of the anti-Npy2r antibody with a synthetic Npy2r-peptide antigen completely abolished the Npy2r staining in the cilia (Fig 5L).

**Fig 6:**
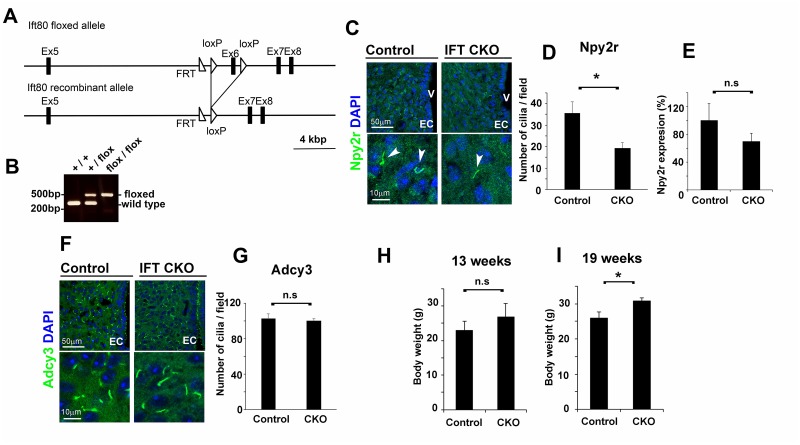
*Ift80 Npy2r-Cre* CKO mice exhibited body weight increase. **A**) Strategy for the conditional deletion of *Ift80*. The *Ift80* exon 6 was deleted by Cre-mediated recombination, resulting in an *Ift80*-null allele. **B**) PCR products of 248 bp or 466 bp were amplified from wild type or floxed *Ift80* alleles, respectively. **C, D**) Cilia in the hypothalamic arcuate nucleus in control and *Ift80 Npy2r-Cre* CKO brains were immunostained with an anti-Npy2r antibody (C). The number of stained cilia/field (25,600 μm^2^) is indicated. The number of the anti-Npy2r antibody-stained cilia (arrowheads) significantly decreased in the hypothalamic arcuate nucleus in the *Ift80 Npy2r-Cre* CKO brain (D) (n = 3; * *p*<0.03). Nuclei were stained with DAPI (blue). **E**) The *Npy2r* mRNA expression levels were measured by Q-PCR in the control and *Ift80 Npy2r-Cre* CKO mouse hypothalamuses (n = 3). **F, G**) Cilia in the hypothalamic arcuate nucleus in control and *Ift80 Npy2r-Cre* CKO brains were immunostained with an anti-Adcy3 antibody (green in F). The number of immunostained cilia/field (25,600 μm^2^) is indicated. The number of cilia immunostained with the anti-Adcy3 antibody was statistically unaltered between control and *Ift80 Npy2r-Cre* CKO mice (G) (n = 3). **H, I**) *Ift80 Npy2r-Cre* CKO mice showed an increased body weight at 19 weeks. Body weights of 13- (H) or 19-week-old (I) male *Ift80 Npy2r-Cre* CKO mice were measured. Body weights of *Ift80 Npy2r-Cre* CKO mice were statistically unaltered at 13 weeks and significantly increased at 19 weeks compared with those of control mice (control, n = 6; CKO, n = 4; *p<0.03). EC: ependymal cells; V: third ventricles. Scale bars, 50 μm (the upper panels in C, F) and 10 μm (the lower panels in C, F).

We then generated *Ift80* flox mice, in which exon 6 of the *Ift80* gene was flanked by two *loxP* sites (Fig 6A). We confirmed that PCR products of 248 bp or 466 bp were amplified from the wild type or floxed *Ift80* allele, respectively (Fig 6B). Deletion of this exon results in a null allele of *Ift80*. Ift80 is a component of the intraflagellar transport protein complex B and is essential for ciliogenesis [62]. In humans, hypomorphic mutations in *IFT80* cause a ciliopathy called asphyxiating thoracic dystrophy or Jeune syndrome [62]. We mated *Ift80* flox mice with BAC-Npy2r-Cre transgenic mice to generate *Ift80 Npy2r-Cre* conditional knockout (CKO) mice to disrupt cilia in *Npy2r*-expressing cells.

*Ift80 Npy2r-Cre* CKO mice were viable and showed no obvious defects compared to control mice before one month of age (1M). To confirm the ciliary defect in *Ift80 Npy2r-Cre* CKO mice, we immunostained the hypothalamic arcuate nucleus with the anti-Npy2r antibody in control and *Ift80 Npy2r-Cre* CKO mice (Fig 6C). We counted the numbers of the cilia stained with the anti-Npy2r antibody in both the control and *Ift80 Npy2r-Cre* CKO mice (Fig 6D). We found that the number of Npy2r-stained cilia markedly decreased in *Ift80 Npy2r-Cre* CKO mice. We examined the *Npy2r* mRNA expression level by Q-PCR in the hypothalamus of the control and *Ift80 Npy2r-Cre* CKO mice, and found no significant change of the *Npy2r* mRNA expression level between them (Fig 6E). We also immunostained cilia with the anti-Adcy3 antibody in the hypothalamic arcuate nucleus in control and *Ift80 Npy2r-Cre* CKO mice (Fig 6F). We found that the number of cilia stained with the anti-Adcy3 antibody in the hypothalamic arcuate nucleus was statistically unaltered between control and *Ift80 Npy2r-Cre* CKO mice (Fig 6G). These results suggest that the cilia of *Npy2r*-expressing cells were specifically affected in *Ift80 Npy2r-Cre* CKO mice in the hypothalamic arcuate nucleus. Even in the control mice, the total number of Npy2r-positive cilia was much lower than that of Adcy3-positive cilia in the arcuate nucleus (35.7±5.1/field for Npy2r, 102.7±9.9/field for Adcy3). No alteration in the Adcy3-positive cilia number in the *Ift80 Npy2r-Cre* CKO mice was detected, probably due to the much larger number of Adcy3-positive cilia compared with that of Npy2r-positive cilia.

Then, we measured body weights of control and *Ift80 Npy2r-Cre* CKO mice (Fig 6H and 6I). We observed no significant change of body weight between control and CKO mice at 13 weeks (Fig 6H). However, we found that body weights of *Ift80 Npy2r-Cre* CKO mice significantly increased by 15% compared to that of control mice at 19 weeks (Fig 6I). This result suggests that cilia in *Npy2r*-expressing cells are essential for normal body weight control in mice.

In the current study, we performed a screen of 138 non-odorant GPCRs to identify GPCRs that localize to cilia in NIH3T3 cells. We found that PRLHR, NMUR1, NPFFR1, and DRD2S localize to the cilia. Interestingly, all of these GPCRs and/or their known ligands have been reported to regulate mammalian feeding behavior, energy homeostasis and/or obesity. Finally, we found that disruption of cilia in *Npy2r*-expressing cells increased body weight. Although the biological importance of Npy2r-positive cilia has been shown in the conventional knockout mice lacking a component of the BBSome complex [57], to the best of our knowledge, *Ift80 Npy2r-Cre* CKO mice are the first mutant mice in which cilia expressing a specific GPCR are disrupted. Thus, using a different approach from previous reports, the current study supports the biological importance of Npy2r-positive cilia in the regulation of body weight.

## Supporting Information

S1 Fig Transfection efficiency of GPCR expression constructs in NIH3T3 cells and Western blot analysis of GPCR transfected in HEK293 cells. **A)** Transfection efficiencies of GPCR expression constructs into NIH3T3 cells were measured. We transfected FLAG-tagged GPCR expression constructs into NIH3T3 cells. After transfection, cells were fixed and immunostained with the anti-FLAG antibody and DAPI. We counted the numbers of FLAG-positive cells and nuclei stained with DAPI to calculate transfection efficiency. The average values from three independent transfection experiments are shown. **B**) Western blot analysis of HEK293 cells transfected with FLAG-tagged GPCR expression constructs. Predicted molecular seizes of GPCRs are as follows: DRD2S, 47.3kDa; DRD2L, 50.6kDa; NPFFR1, 47.8kDa NMUR1, 47.4kDa; NPFFR2, 47.4kDa; NMUR2, 47.8kDa; PRLHR, 41.1kDa.
(EPS)

S1 Table GPCRs whose subcellular localizations were determined in NIH3T3 cells. Constructs expressing a FLAG- or mCherry-tagged full length GPCR under the control of the CAG promoter were generated. Each of these constructs was transfected into NIH3T3 cells. Ciliary localization was determined by immunofluorescent staining using the anti-FLAG or anti-mCherry antibody together with the anti-acetylated alpha-tubulin antibody to label the ciliary axoneme.
(XLS)
